# Binary Host‐induced Exciplex Enabled High Color‐Rendering Index of 94 for Carbon Quantum Dot‐Based White Light‐Emitting Diodes

**DOI:** 10.1002/advs.202404485

**Published:** 2024-06-13

**Authors:** Renjing Chen, Zhibin Wang, Qian Teng, Chenhao Li, Jinsui Li, Lingwei Zeng, Ruidan Zhang, Feng Huang, Lei Lei, Fanglong Yuan, Daqin Chen

**Affiliations:** ^1^ College of Physics and Energy Fujian Normal University Fujian Provincial Key Laboratory of Quantum Manipulation and New Energy Materials Fuzhou 350117 P. R. China; ^2^ Key Laboratory of Theoretical & Computational Photochemistry of Ministry of Education College of Chemistry Beijing Normal University Beijing 100875 P. R. China; ^3^ School of Chemistry and Chemical Engineering Hunan University of Science and Technology Xiangtan 411201 P. R. China; ^4^ Institute of Optoelectronic Materials and Devices China Jiliang University Hangzhou 310018 P. R. China; ^5^ Fujian Provincial Collaborative Innovation Center for Advanced High‐Field Superconducting Materials and Engineering Fuzhou 350117 P. R. China; ^6^ Fujian Provincial Engineering Technology Research Center of Solar Energy Conversion and Energy Storage Fuzhou 350117 P. R. China

**Keywords:** binary host‐induced exciplex, carbon quantum dots, color rendering index, white light‐emitting diodes

## Abstract

White light‐emitting diodes (WLEDs) with high color‐rendering index (CRI, >90) are important for backlight displays and solid‐state lighting applications. Although the well‐developed colloidal quantum dots (QDs) based on heavy metals such as cadmium and lead are promising candidates for WLEDs, the low CRI still remains a significant limitation. In addition, the severe toxicity of heavy metals greatly limits their widespread use. Herein, the study demonstrates low‐cost and environmentally friendly carbon quantum dots (CQDs)‐based WLEDs that exhibit a high CRI of 94.33, surpassing that of conventional cadmium/lead‐containing QD‐based WLEDs. This achievement is attained through the employment of a binary host‐induced exciplex strategy. The high hole/electron mobility and suitable energy levels of the donor and acceptor give rise to a broadband orange–yellow emission stemming from the exciplex. As the host, the binary exciplex is capable of contributing blue and orange–yellow emission components while efficiently mitigating the aggregation‐induced quenching of CQDs. Meanwhile, CQDs effectively address the deep‐red emission gap, enabling the realization of CQDs‐based WLEDs with high CRI. These WLEDs also exhibit a remarkably low turn‐on voltage of 2.8 V, a maximum luminance exceeding 2000 cd m^−^^2^, a correlated color temperature of 4976 K, and Commission Internationale de l'Eclairage coordinates of (0.34, 0.32).

## Graphical abstract

High‐performance carbon quantum dots‐based white light‐emitting diodes with impressive high color rendering index of 94.33 are prepared by well‐designed binary host‐induced exciplex host structure. The resulting devices exhibited Comission Internationale de l'Eclairage coordinates of (0.34, 0.32) and a *L*_max_ of 2375.5 cd m^−2^.

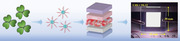

## 1 Introduction

Quantum dots (QDs) are emerging as promising materials for the next generation of lighting and displays owing to their excellent luminescent characteristics such as high quantum yield, tunable spectrum, and high color purity. White light‐emitting diodes (WLEDs) play a pivotal role as a significant lighting source in various applications, including flat panel displays,^[^ ^1^, ^2^ ^]^ industrial lighting,^[^ ^3^ ^]^ automotive lamps,^[^ ^4^ ^]^ surgical table lamps,^[^ ^5^ ^]^ and more. Furthermore, the color‐rendering index (CRI) is a vital performance metric for WLEDs, within specialized domains like medicine, art, and design, precise color perception is imperative, demanding a CRI surpassing 90.^[^ ^6^, ^7^ ^]^ To fulfill the lighting requirements of these special areas, therefore, it is crucial to improve the CRI value. **Table** **1** provides an overview of the key parameters associated with QDs‐based WLEDs. Conventional Cd^2^⁺‐based semiconductor QDs and perovskite‐based WLEDs have achieved impressive external quantum efficiency (EQE) exceeding 10%. Nevertheless, these QDs face the problem of reabsorption, narrowing the spectrum of the WLEDs and consequently leads to a low CRI value.^[^ ^8^ ^]^ Not only that, the presence of heavy metal elements like Cd^2^⁺ and Pb^2^⁺ hinders the long‐term sustainability and applications of these materials in future lighting and display technologies.^[^ ^9^, ^10^ ^]^ Therefore, to better align with the discerning requirements of lighting quality, developing a new generation of luminescent QD materials with lower toxicity and cost for the fabrication of WLEDs with high CRI is highly desired.

**Table 1:** Comparison of the CRI in WLEDs fabricated with Cd/Pb‐based QDs and nontoxic CQDs.

Materials category	Examples	Toxicity	CRI	CCT [K]	V_on_ [V]	Reference
Cd‐based quantum dots	CdSe/ZnS	Yes	NA	NA	3.2	[39]
	CdSe/ZnS/P(VDF‐TrFE)	Yes	NA	6500	2.7	[40]
	P(VDF‐TrFE)/CdSe/ZnS	Yes	NA	25 000	2.6	
	InZnP/ZnSe/ZnS: InZnP/ZnSeS: CdZnS/ZnS	Yes	98	5003	NA	[41]
Pb‐based quantum dots	CsPb(Br_x_/I_3‐x_):MEH:PPV	Yes	NA	NA	3.3	[42]
	α‐CsPbI_3_:δ‐CsPbI_3_	Yes	NA	NA	3.6	[43]
Pb‐based perovskite nanocrystal	PBABr_1.4_(Cs_0.7_FA_0.3_PbBr_3_): CsPbBrI_2_	Yes	69	5500	NA	[44]
Cd/Pb‐free carbon quantum dots	CQDs:PVK	No	83	7694	NA	[12]
	**CQDs:PO‐T2T:PTAA**	**No**	**94.33**	**4976**	**2.8**	This work
	CQDs:PO‐T2T:TFB	No	85.32	5503	4.6	This work
	CQDs:PO‐T2T:Poly‐TPD	No	90	3687	4.6	This work

Note: 0 < x < 1.

In recent years, carbon quantum dots (CQDs) have emerged as a promising luminescent material for LED display and lighting applications due to their tunable and excellent optical properties, low toxicity, low manufacturing cost, and diverse precursor sources.^[^ ^11^, ^12^ ^]^ So far, CQDs‐based LEDs have garnered significant research investment, leading to their development across the entire visible spectrum, including blue to red, and white light emission.^[^ ^13^, ^14^, ^15^, ^16^, ^17^, ^18^, ^19^, ^20^, ^21^, ^22^, ^23^, ^24^ ^]^ Currently, CQDs‐based WLEDs fall into two main categories: yellow CQDs phosphors are coated onto a blue LED chip, or combined with red and green phosphors and coated onto an ultraviolet chip, for photoluminescent excitation; CQDs serve as the emitting layer (EML), fabricated into electroluminescent (EL) devices using a spin‐coating method.^[^ ^25^, ^26^, ^27^, ^28^ ^]^ The EL device offers several advantages for future LED development, including a simpler structure, reduced size, and extended service life, eliminating the need for an additional excitation light source.

Currently, the challenge of addressing aggregation‐induced quenching (AIQ) in solid films remains unresolved for single‐component ultra‐broadband white CQDs. Consequently, the prepared WLEDs exhibit weak or no electroluminescence, leading to significantly inferior device performance. The host‐guest doping strategy effectively addresses the challenge of AIQ by dispersing the CQDs within organic semiconductor materials.^[^ ^29^, ^30^, ^31^, ^32^, ^33^, ^34^ ^]^ Currently, the preparation of CQDs‐based WLEDs primarily relies on the mainstream method of combining wide‐bandgap host materials, such as poly(9‐vinylcarbazole) (PVK) and poly(9,9‐dioctylfluorenyl‐2,7‐diyl)‐alt‐(4,4′‐(N‐(4‐butylphenyl)) (TFB), with orange–yellow CQDs as the EML.^[^ ^35^, ^36^, ^37^, ^38^ ^]^ Although the overall performance of the device has experienced remarkable enhancements, the absence of a deep‐red component hinders the achievement of a high CRI and color fidelity, which are essential for vibrant color displays and high saturation. Consequently, addressing this deficiency in the deep‐red component is crucial for meeting market demands and attaining a high CRI.

Herein, we demonstrated CQDs‐based WLEDs with high CRI of 94 via a binary host‐induced exciplex strategy. The adopted CQDs synthesized via a solvothermal process, utilizing natural green plants as the carbon source exhibit a prominent deep‐red photoluminescence (PL) emission peak at 675 nm, with an ultra‐narrow FWHM of merely 19 nm. The binary host‐induced exciplex strategy was employed with poly[bis(4‐phenyl)(2,4,6‐trimethylphenyl)amine] (PTAA), TFB, or [poly(bis(4‐phenyl)(4‐butylphenyl)amine)] (Poly‐TPD) as a donor, and with [2,4,6‐Tris[3‐(diphenylphosphinyl) phenyl]−1,3,5‐Triazine] (PO‐T2T) as electron acceptor, respectively. When employing PTAA:PO‐T2T as the binary host, this approach efficiently generates blue and orange–yellow light emissions. When combined with the deep‐red emitting CQDs, it ultimately culminates in the successful achievement of WLEDs featuring a high CRI. Consequently, the CQDs‐based WLEDs, utilizing PTAA and the small‐molecule PO‐T2T as a binary host‐induced exciplex, exhibit remarkable optoelectronic properties, including a low turn‐on voltage (*V*_t_) of 2.8 V, a CRI of up to 94.33, a *L*_max_ exceeding 2000 cd m^−2^, a CCT of 4976 K, and CIE coordinates of (0.34, 0.32). This work provides a new development path for future realization of CQDs‐based WLEDs with cost‐effective, high‐CRI, and high‐brightness.

## 2 Results and Discussion

**Figure 1:**
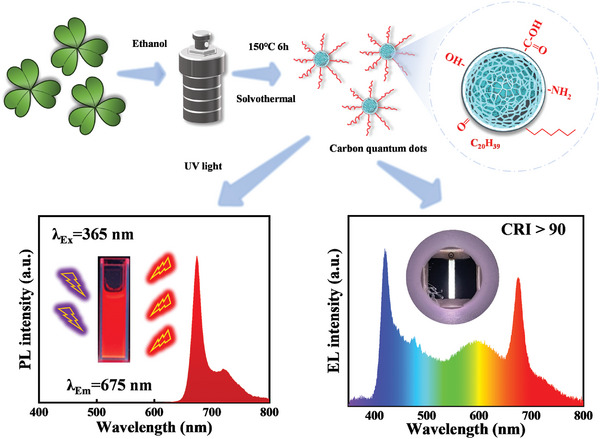
Schematic diagram of the synthesis process, optical property, and EL application of CQDs.

The emission of CQDs in deep‐red region is beneficial for enhancing the CRI of WLEDs, prompting us to prioritize the synthesis of CQDs exhibiting deep‐red emission. As illustrated in **Figure** **1**, CQDs were synthesized through a solvothermal method utilizing oxalis corniculata L., and anhydrous ethanol as precursor materials. These precursors were subsequently transferred to a 25 mL autoclave lined with polytetrafluoroethylene, where they underwent a 6 h reaction at 150 °C. The resulting crude product was subjected to purification through a silica gel column chromatography process to yield deep‐red CQDs with surficial functional groups. The exciplexes we have developed exhibit broadband emissions, with emission peaks centered in the orange–yellow region. By integrating these CQDs into EL devices, we can compensate for the absence of deep‐red light in current CQDs‐based WLEDs, ultimately achieving a high CRI for CQDs‐based WLEDs.

**Figure 2:**
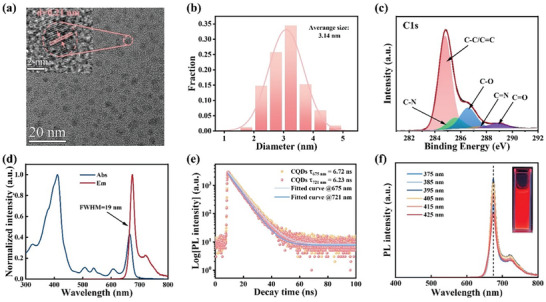
a) Transmission electron microscopy (TEM) images of CQDs. Inset is a high‐resolution TEM (HRTEM) image. b) Diameter histogram of the CQDs. c) High‐resolution C1s X‐ray photoelectron spectroscopy (XPS) spectrum of CQDs. d) The PL and ultraviolet–visible (UV–vis) absorption spectrum of CQDs. e) The PL decay curves of CQDs at 675 and 721 nm with an excitation wavelength of 375 nm. f) PL spectra of CQDs excited at various wavelengths. Inset is photographs of the CQDs solution under 365 nm UV.

The TEM measurements confirmed the well‐dispersed quasi‐spherical nanoparticle nature of the CQDs (**Figure** **2**a; Figure S1a, Supporting Information). Furthermore, the HRTEM mage, displayed in the inset of Figure 2a, enabled the estimation of lattice spacing using fast Fourier transform (FFT) calculations. The resulting lattice spacing was determined to be 0.21 nm, in line with the in‐plane (100) lattice spacing characteristic of graphene (Figure S1b, Supporting Information). These findings are consistent with prior research conducted on CQDs derived from taxus leaves and mulberry leaf sources.^[^ ^38^, ^45^ ^]^ As depicted in Figure 2b, the size distribution of the CQDs was determined by enumerating ≈400 particles (Figure S1c, Supporting Information). These particles exhibited sizes within the range of 1.9 to 5.4 nm, with an average size of 3.14 nm. The X‐ray powder diffraction (XRD) pattern of CQDs shows a wide diffraction peaks at 23° (Figure S2, Supporting Information), corresponding to the (002) of graphite.^[^ ^46^, ^47^, ^48^ ^]^

The XPS analysis revealed that the composition of the CQDs primarily consists of the elements C, O, and N, with atomic ratios of 74.22%, 23.1%, and 2.68%, respectively (Figure S3a, Supporting Information). High‐resolution XPS spectra of the C1s exhibited distinct peaks corresponding to C─C/C═C (284.8 eV), C─N (285.6 eV), C─O (286.6 eV), C═N (287.4 eV), and C═O (288.9 eV) (Figure 2c). The N1s spectra at 397.9, 400.3, and 402.6 eV displayed three peaks, attributed to pyridine N, pyrrolic N, and graphitic N, respectively (Figure S3b, Supporting Information). Additionally, the O1s spectra indicated the presence of C═O and C─O, with binding energies of 532.2 and 533.7 eV, respectively (Figure S3c, Supporting Information).^[^ ^49^ ^]^ The fourier transform infrared spectroscopy (Figure S4, Supporting Information) shows that the characteristic peak at 2920 cm^−1^ corresponds to the stretching vibration of C─H, indicating the presence of long alkyl chains on the surface of CQDs. In addition, characteristic peaks were observed at 1630 cm^−1^, corresponding to the stretching vibration of C═C, indicating that CQDs has large conjugated systems. 1375 and 1079 cm^−1^ are attributed to the stretching vibration of C─N and the stretching vibration of C─O─C respectively. In addition, the characteristic peak at 745 cm^−1^ indicates the presence of a polymer shell.^[^ ^38^, ^45^ ^]^ As shown in the Figure S5 (Supporting Information), there are two peaks in the Raman spectrum, 1597 and 1350 cm^−1^, corresponding to the G and D bands respectively. The strength ratio of G and D bands is 1.23, which indicates that CQDs has good crystallinity.^[^ ^37^ ^]^ Figure 2d illustrates the UV–vis absorption and fluorescence spectra of CQDs dissolved in an ethanol solution. The spectra display a broad absorption band spanning from 300 to 700 nm. The absorption peaks at 411 and 665 nm are attributed to the Soret band and Q band of porphyrin molecules, respectively.^[^ ^50^, ^51^ ^]^ The emission peak intensity reaches its maximum at 675 nm when excited with a wavelength of 395 nm, with an exceptionally narrow FWHM of 19 nm. The narrow FWHM could potentially be attributed to a combination of weak electron‐phonon coupling and high structural rigidity.^[^ ^14^, ^21^ ^]^ Additionally, a shoulder peak at 721 nm was detected, attributed to the inter‐ or intra‐ring stretching mode of C─C, a characteristic feature in polycyclic aromatic hydrocarbon systems.^[^ ^49^, ^52^ ^]^

To gain insight into the carrier recombination dynamic process of CQDs, time‐resolved PL (TRPL) spectra were conducted (Figure 2e). The lifetimes of the PL signals at 675 and 721 nm were monitored with excitation using a 375 nm laser and estimated to be 6.72 and 6.23 ns, respectively. The fitting with a single‐exponential decay function and the short lifetimes suggests that excitons within CQDs predominantly participate in radiative recombination, leading to single exciton emission. Moreover, the similar fluorescence lifetimes indicate the presence of a consistent luminescence center, consistent with previous reports.^[^ ^38^ ^]^

Furthermore, with a varying excitation wavelength, the PL spectra of CQDs consistently remained at 675 nm, showing no variation with the excitation wavelength. When CQDs were dissolved in an ethanol solution, they exhibited a pronounced deep‐red emission when excited by UV (Figure 2f) with a photoluminescence quantum yields (PLQYs) of 32.18% (Figure S6, Supporting Information). Monitoring the excitation spectrum obtained at the emission wavelength of 675 nm revealed that the emission peak intensity was strongest at the excitation wavelength of 395 nm, consistent with the PL spectra observed at different excitation wavelengths (Figure S7, Supporting Information).

**Figure 3:**
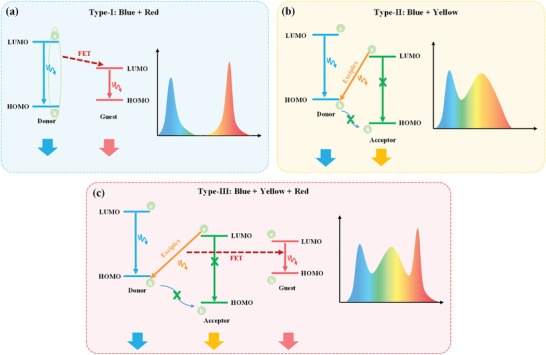
Schematic diagram depicting the design of the device energy levels. a) Type‐I: Host‐guest system. b) Type‐II: Exciplex system. c) Type‐III: Co‐host system.

The CRI is widely acknowledged as a crucial metric for evaluating the ability of WLEDs in accurately reproducing the colors of illuminated objects. In our study, we devised three distinct device structures aimed at realizing CQDs‐based WLEDs with elevated CRI and brightness. **Figure** **3**a illustrates a Type‐I device structure, employing a single host strategy where a large bandgap material transfers partial energy to CQDs via Förster energy transfer (FET), resulting in a blue emission from host and a red emission from CQDs.^[^ ^53^ ^]^ While many CQDs‐WLEDs rely on the single host strategy, it is not suitable for our case due to the lack of orange–yellow components.^[^ ^26^, ^54^ ^]^ Additionally, most single host materials primarily act as electron donors, potentially resulting in excess hole injection and insufficient electron injection. In addressing the issue of hole accumulation, we incorporated electron transport materials to achieve balanced charge transport within the host‐guest system. This facilitated exciplex formation, resulting in a new peak in the yellow–orange light region. The exciplex effectively compensates for the absence of the orange–yellow component, enhancing charge carrier injection efficiency, reducing electron and hole injection barriers, and ultimately resulting in a lower device *V*_t_ and improved luminous efficiency. This approach paves the way for the realization of high‐performance CQDs‐based WLEDs (Figure 3b).^[^ ^55^ ^]^ In Type II, exciplex formation involves an intermolecular charge transfer from the lowest unoccupied molecular orbital (LUMO) of the acceptor to the highest occupied molecular orbital (HOMO) of the donor. This process requires high carrier mobility for both electron and hole transport materials, along with matched energy levels. The energy level difference between the HOMO and LUMO should be sufficiently large (generally not <0.4 eV) to facilitate the aggregation of electrons and holes at the interfaces of heterojunction, forming the excited state of the exciplex.^[^ ^56^, ^57^ ^]^ In Type‐III (Figure 3c), a binary host‐induced exciplex serves as the host for energy transfer to CQDs. This strategy effectively addresses the absence of the yellow and orange emission region and mitigates the injection barrier for electrons and holes. Consequently, this reduces the *V*_t_ of the device and enhances luminous efficiency, offering a promising avenue for high‐performance CQDs‐based WLEDs.

**Figure 4:**
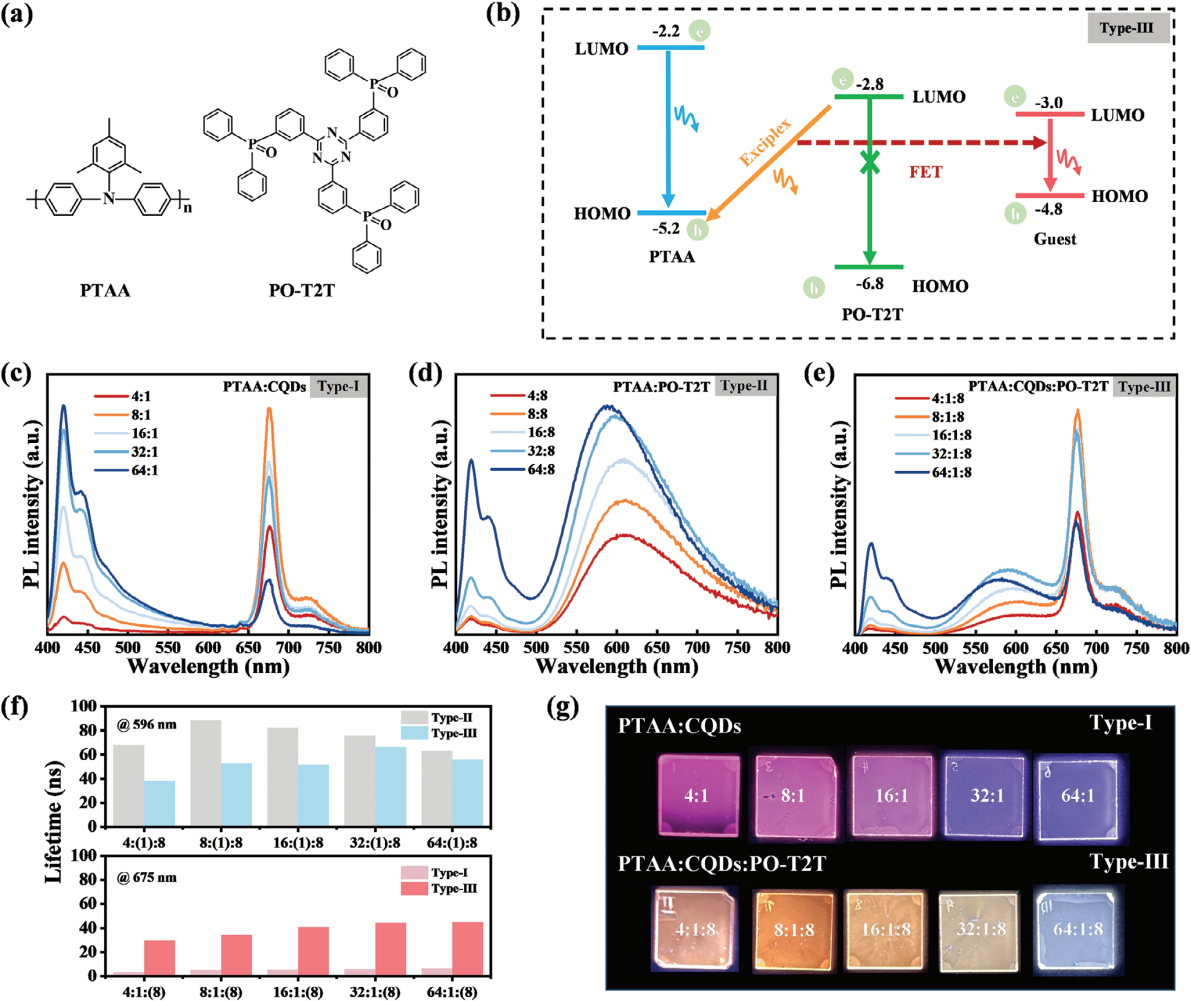
a) The molecular structures of PTAA and PO‐T2T. b) The diagram illustrates the energy transfer process. The PL spectra of c) Type‐I (PTAA:CQDs), d) Type‐II (PTAA:PO‐T2T), and e) Type‐III (PTAA:CQDs:PO‐T2T) films. f) TRPL spectra of Type‐I, Type‐II and Type‐III thin films at various proportions. g) The thin‐film color of Type‐I and Type‐III at various proportions.

In order to verify the rationality of the device design, spectral analysis of the thin films in the device architecture was conducted. First, the aniline compound PTAA, renowned for its high hole mobility ranging from ≈10^−3^ to 10^−2^ cm^2^ V^−1^ S^−1^, was chosen as the donor. Simultaneously, the triazine derivative electron transport material PO‐T2T, with an electron mobility of ≈10^−3^ cm^2^ V^−1^ S^−1^, was selected as the acceptor. This selection is justified by the tendency of aniline‐based donor materials to form exciplex with triazine‐derived acceptor materials, owing to their high and closely matched carrier mobility (**Figure** **4**a).^[^ ^55^, ^56^, ^58^, ^59^ ^]^ The LUMO and HOMO energy levels of the exciplex are −2.8 and −5.2 eV, respectively (Figure 4b, Type‐III).^[^ ^60^, ^61^ ^]^ The energy gap of the CQDs is only 1.8 eV, with both its HOMO and LUMO levels situated within the exciplex. The absorption spectrum of CQD overlaps with the emission spectrum of the host materials, which is the premise of FET (Figure S8, Supporting Information). By regulating the doping concentration of CQDs, the distance between CQDs and the host material molecules exceeds the effective distance for FET (≈10 nm). This leads to incomplete energy transfer, resulting in the emission of both exciplex and CQDs. Moreover, the shallow LUMO of PTAA and deep HOMO of PO‐T2T play a pivotal role in preventing exciton leakage from the CQDs, effectively impeding exciton diffusion.

To confirm that FET can be controlled by adjusting the concentration of CQDs, PL spectra of films prepared with various structures were described (Figure 4c–e), and the host‐guest system of PTAA:CQDs, the exciplex of PTAA:PO‐T2T, and the co‐host strategy of PTAA:CQDs:PO‐T2T were labeled as “Type‐I,” “Type‐II,” and “Type‐III,” respectively. The results demonstrate that the desired spectrum can be achieved by carefully adjusting the concentration of CQDs. As anticipated, various device architectures are expected to exhibit distinct emission peaks. As shown in the Figure 4c, reducing the concentration of PTAA increases the relative content of CQDs, resulting in enhanced emission intensity of the CQDs. Notably, within PTAA volume ratios from 4:1 to 64:1, the AIQ effect of the CQDs is not predominant. Therefore, the intensity of red‐light emission is primarily correlated with the CQD content. In Figure 4d, the broad yellow–orange emission band originates from the exciplex formed between PTAA and PO‐T2T, rather than from the emission peak of PO‐T2T itself. Consequently, the interaction between PTAA and PO‐T2T results in exciplex emission. Additionally, when the proportion of PTAA is increased, emission from PTAA ≈420 nm is also observed. Notably, the broad spectrum exhibited by Type‐III suggests its potential to enable high‐CRI CQDs‐based WLEDs (Figure 4e). To further validate the universality of the device architecture, we selected two prevalent hole‐transport materials for investigation, namely, TFB (hole mobility: ≈10^−4^ cm^2^ V^−1^ S^−1^) and Poly‐TPD (hole mobility: ≈10^−4^ cm^2^ V^−1^ S^−1^), for comparative analysis (Figure S9, Supporting Information).^[^ ^62^, ^63^ ^]^ Figure S10 (Supporting Information) illustrates the corresponding PL spectra of thin films of the host‐guest strategy of TFB:CQDs, the exciplex of TFB:PO‐T2T, and the co‐host strategy of TFB:CQDs:PO‐T2T. The exciplex peaks produced by TFB and PO‐T2T appear at ≈540 nm. Compared with those produced by PTAA and PO‐T2T, the peaks of these exciplex blue‐shifted. This may be attributed to the deeper HOMO energy level of TFB compared to that of PTAA, and the larger exciton binding energy formed by TFB compared to that of PTAA, resulting in a shorter emission wavelength.^[^ ^64^ ^]^ Figure S11 (Supporting Information) shows the PL spectra of the host‐guest strategy of Poly‐TPD:CQDs, the exciplex of Poly‐TPD:PO‐T2T, and the co‐host strategy of Poly‐TPD:CQDs:PO‐T2T. Interestingly, the Poly‐TPD:PO‐T2T system exhibits a similar spectral shape of PL spectra as the PTAA:PO‐T2T system, probably due to their analogous molecular structures and energy levels. The absorption spectra depicted in Figure S12 (Supporting Information) for the three device architectures exhibit no discernible new peaks, indicating the absence of fresh exciplex formation or aggregation.^[^ ^65^ ^]^

The TRPL measurements were conducted on the three types of films, as illustrated in Figure 4f and Figure S8 (Supporting Information). At a probing wavelength of 596 nm (originating from the exciplex), the PL liftetimes of PTAA:PO‐T2T at various ratios ranged from 68 to 88.6 ns, notably longer than those of PTAA:CQDs:PO‐T2T (38.4–66.2 ns), indicating the presence of FET from the exciplex to the CQDs. At a probing wavelength of 675 nm (originating from the CQDs), the PL lifetimes of co‐host:CQDs ranged from 29.9 to 44.9 ns, significantly longer than those of PTAA:CQDs (3.4–6.5 ns), suggesting enhanced FET efficiency with the co‐host strategy. Additionally, Figure S13 (Supporting Information) presents the PLQYs for the three types of films. Notably, none of the films exhibited significantly high PLQYs upon transitioning to solid‐state, likely attributable to AIQ. Therefore, addressing the issue of AIQ, a crucial determinant for enhancing the PLQY of solid films and device efficiency, remains an avenue ripe for future exploration. We conducted measurements of the morphology of PTAA, PO‐T2T, PTAA:PO‐T2T, and PTAA:CQDs:PO‐T2T films, along with their root‐mean‐square (RMS) values, which were found to be 1.25, 0.54, 1.13, and 1.56 nm, respectively (Figure S14, Supporting Information). The results show that the roughness of the film does not change much after adding CQDs. Different ratios of Type‐I and Type‐III resulted in films of varying colors (Figure 4g). It is evident that the PTAA:CQDs:PO‐T2T combination, at a mass ratio of 32:1:8, produces a warm white light emission film upon UV excitation, indicating its promising potential for the development of CQDs‐based WLEDs.

**Figure 5:**
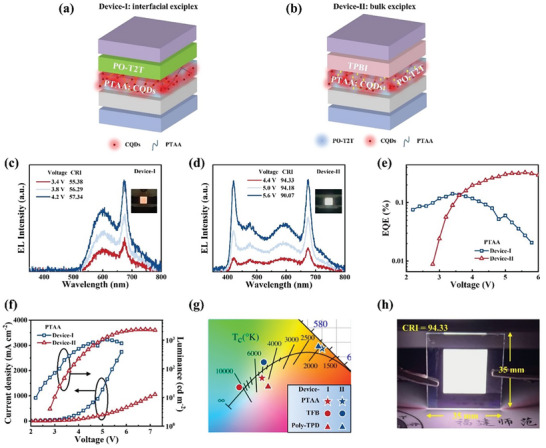
Device structure of exciplex system. a) Interfacial exciplex. b) Bulk exciplex. c) The EL spectra of Device‐I at different voltages. d) The EL spectra of Device‐II at different voltages. e) The EQE‐voltage curve of Device‐I and Device‐II. f) Luminance–voltage–current density (L‐V‐J) curve of Device‐I and Device‐II. g) The CIE coordinates of the Device‐I and Device‐II, and the CIE coordinates of the TFB and Poly‐TPD as donors respectively. The shape in the figure represents the type of donor, with the shape filling color indicating Device‐I (blue) and Device‐II (red), respectively. h) Photograph of operating large‐area Device‐II.

To assess the effectiveness of incorporating exciplexes in fabricating high‐CRI CQDs‐based WLEDs, we constructed two distinct device structures that favor exciplex formation. The first structure is the interfacial exciplex (**Figure** **5**a), wherein the donor and acceptor are deposited separately through vacuum thermal evaporation. This process leads to the formation of excitons at the interface where these materials come into contact. The second approach involves bulk exciplex formation (Figure 5b), where the donor and acceptor are mixed using a solution‐processed method. In comparison to the interfacial exciplex, bulk exciplex may exhibit enhanced intermolecular interactions.^[^ ^66^ ^]^ We labeled the interfacial exciplex as Device‐I, comprising an ITO anode, a poly(2,3‐dihydrothiophene‐1,4‐diinyl)‐poly(styrene sulfonate) (PEDOT:PSS) hole injection layer, a PTAA:CQDs mixed emissive layer, a PO‐T2T electron transport layer, and a LiF‐coated aluminum cathode. In contrast, Device‐II, designated as the bulk exciplex, includes an ITO anode, a PEDOT:PSS hole injection layer, a PTAA:PO‐T2T:CQDs mixed emissive layer, a 1,3,5‐tris(2‐n‐phenylbenzimidazolyl)‐benzene (TPBi) electron transport layer, and a LiF‐coated cathode. The EL spectra of Device‐I display a broadband orange–yellow emission with peaks centered at 598 and 675 nm under various applied voltages (Figure 5c). The inset displays a uniform orange–yellow emission in CQDs‐LEDs based on the Device‐I, attributed to the absence of a blue light component from PTAA. Conversely, the EL spectra of CQDs‐LEDs based on Device‐II at various bias voltages exhibit a favorable pure white emission, and the CRI is all above 90, with excellent spectral stability and peaks at 421, 598, and 675 nm (Figure 5d). Operated at a voltage of 5 V, Device‐II exhibits a remarkable high CRI of 94.33, marking the highest value reported for CQDs‐based WLEDs (refer to Table S1, Supporting Information). The performance of Device‐I and II is showed in Figure 5e,f. Device‐I possesses an ultra‐low *V*_t_ of 2.2 V, an EQE of 0.14%, and a brightness of 1034 cd m^−2^. The Device‐II emits a vivid and pure white light with an EQE_max_ of 0.32%, an *L*_max_ of 2375.5 cd m^−2^, a CCT of 4976 K, and CIE coordinates of (0.3429, 0.3214) (Figure 5g). In addition, we fabricated the first reported CQDs‐based WLEDs with a large‐area of 20 × 20 mm^2^ (Figure 5h), demonstrating uniform and bright emission. These findings strongly indicate the significant potential of the exciplex system in the development of CQDs‐based WLEDs.

Furthermore, EL devices employing two other donors, TFB and Poly‐TPD, were fabricated, and the EL spectra along with their device performance characteristics are depicted in Figures S15 and S16 (Supporting Information). Notably, the bulk exciplex all showed white light at different color temperatures, and the interfacial exciplex of TFB also showed white light, while the interfacial exciplex of Poly‐TPD showed similar results to PTAA, but not as good as PTAA in all aspects. The stability test of WLED was carried out, as shown in Figure S17 (Supporting Information). When the initial brightness is 100 cd m^−2^, the life *T*_50_ of WLED was 2989 s. In terms of device reproducibility, 50 devices were assessed within an unencapsulated N_2_ glove box, resulting in peak brightness and EQE histograms as shown in Figure S18 (Supporting Information). These histograms revealed an average brightness of 1840 cd m^−2^ and an average EQE of 0.23%. These results underscore the robust reproducibility of CQDs‐based WLEDs and demonstrate their potential for solid‐state lighting and display applications.

## 3 Conclusion

In this work, we successfully synthesized ultra‐narrow bandwidth CQDs with a FWHM of 19 nm using a one‐pot solvothermal method. Based on the low‐cost and environmentally friendly CQDs, we demonstrated WLEDs with high CRI of 94.33 via employing a binary host‐induced exciplex strategy for the first time. The binary exciplex is capable of contributing blue and orange–yellow emission components while CQDs effectively address the deep‐red emission gap, enabling the realization of CQDs‐based WLEDs with high CRI. The resulting devices exhibited CIE coordinates of (0.34, 0.32) and a *L*_max_ of 2375.5 cd m^−2^. The binary host‐induced exciplex strategy in this work will pave the way for the development of cost‐effective, high‐CRI, and high‐brightness CQDs‐based WLEDs.

## Conflict of Interest

The authors declare no conflict of interest.

## Supporting information

Supporting Information

## Data Availability

The data that support the findings of this study are available from the corresponding author upon reasonable request.

## References

[1] G. Biwa , A. Aoyagi , M. Doi , K. Tomoda , A. Yasuda , H. Kadota , J. Soc. Inf. Disp. 2021, 29, 435.

[2] R. Wocheng , L. Bingqing , L. Zhaojun , SID Symp. Dig. Tech. Pap. 2021, 52, 605.

[3] S. Wang , D. Su , M. Ma , W. Kuang , Sustainable Prod. Consump. 2021, 27, 1808.

[4] Y. Zhu , L. Guo , Y. Lee , X. Xu , J. Xie , G. Zhang , Y. Hu , SID Symp. Dig. Tech. Pap. 2019, 50, 628.

[5] T. Ide , Y. Kinugawa , Y. Nobae , T. Suzuki , Y. Tanaka , I. Toda , K. Tsubota , Plast. Reconstr. Surg. Glob. Open 2015, 3, e562. 26893987 10.1097/GOX.0000000000000498 PMC4727714

[6] M. C. Gather , A. Köhnen , K. Meerholz , Adv. Mater. 2011, 23, 233. 20976676 10.1002/adma.201002636

[7] Z. Ma , Z. Shi , D. Yang , Y. Li , F. Zhang , L. Wang , X. Chen , D. Wu , Y. Tian , Y. Zhang , L. Zhang , X. Li , C. Shan , Adv. Mater. 2021, 33, 2001367. 10.1002/adma.202001367 33225543

[8] Y. Wei , Z. Cheng , J. Lin , Chem. Soc. Rev. 2019, 48, 310. 30465675 10.1039/c8cs00740c

[9] A. Dey , J. Ye , A. De , E. Debroye , S. K. Ha , E. Bladt , A. S. Kshirsagar , Z. Wang , J. Yin , Y. Wang , L. N. Quan , F. Yan , M. Gao , X. Li , J. Shamsi , T. Debnath , M. Cao , M. A. Scheel , S. Kumar , J. A. Steele , M. Gerhard , L. Chouhan , K. Xu , X. Wu , Y. Li , Y. Zhang , A. Dutta , C. Han , I. Vincon , A. L. Rogach , et al., ACS Nano 2021, 15, 10775. 34137264 10.1021/acsnano.0c08903 PMC8482768

[10] F. P. García De Arquer , D. V. Talapin , V. I. Klimov , Y. Arakawa , M. Bayer , E. H. Sargent , Science 2021, 373, eaaz8541. 34353926 10.1126/science.aaz8541

[11] F. Yuan , T. Yuan , L. Sui , Z. Wang , Z. Xi , Y. Li , X. Li , L. Fan , Z. Tan , A. Chen , M. Jin , S. Yang , Nat. Commun. 2018, 9, 2249. 29884873 10.1038/s41467-018-04635-5 PMC5993800

[12] J. Xu , Y. Miao , J. Zheng , H. Wang , Y. Yang , X. Liu , Nanoscale 2018, 10, 11211. 29873657 10.1039/c8nr01834k

[13] F. Yuan , Z. Wang , X. Li , Y. Li , Z. Tan , L. Fan , S. Yang , Adv. Mater. 2017, 29, 1604436. 10.1002/adma.201604436 27879013

[14] R. Chen , Z. Wang , T. Pang , Q. Teng , C. Li , N. Jiang , S. Zheng , R. Zhang , Y. Zheng , D. Chen , F. Yuan , Adv. Mater. 2023, 35, 2302275. 10.1002/adma.202302275 37228040

[15] X. Wang , B. Wang , H. Wang , T. Zhang , H. Qi , Z. Wu , Y. Ma , H. Huang , M. Shao , Y. Liu , Y. Li , Z. Kang , Angew. Chem., Int. Ed. 2021, 133, 12693. 10.1002/anie.202103086 33754433

[16] T. Yuan , F. Yuan , L. Sui , Y. Zhang , Y. Li , X. Li , Z. Tan , L. Fan , Angew. Chem., Int. Ed. 2023, 62, e202218568. 10.1002/anie.202218568 36924197

[17] B. Zhao , H. Ma , H. Jia , M. Zheng , K. Xu , R. Yu , S. Qu , Z. Tan , Angew. Chem., Int. Ed. 2023, 135, e202301651. 10.1002/anie.202301651 36997339

[18] K. Xu , M. Zheng , H. Ma , B. Zhao , H. Jia , C. Zhang , Z. Ren , C. Li , Z. Tan , Chem. Eng. J. 2023, 470, 144112.

[19] Y. Wang , K. Wang , F. Dai , K. Zhang , H. Tang , L. Wang , J. Xing , Nat. Commun. 2022, 13, 6495. 36310232 10.1038/s41467-022-34291-9 PMC9618563

[20] F. Yuan , T. Yuan , L. Sui , Z. Wang , Z. Xi , Y. Li , X. Li , L. Fan , Z. Tan , A. Chen , M. Jin , S. Yang , Nat. Commun. 2018, 9, 2249. 29884873 10.1038/s41467-018-04635-5 PMC5993800

[21] F. Yuan , Y.‐K. Wang , G. Sharma , Y. Dong , X. Zheng , P. Li , A. Johnston , G. Bappi , J. Z. Fan , H. Kung , B. Chen , M. I. Saidaminov , K. Singh , O. Voznyy , O. M. Bakr , Z.‐H. Lu , E. H. Sargent , Nat. Photonics 2020, 14, 171.

[22] X. Zhang , Y. Zhang , Y. Wang , S. Kalytchuk , S. V. Kershaw , Y. Wang , P. Wang , T. Zhang , Y. Zhao , H. Zhang , T. Cui , Y. Wang , J. Zhao , W. W. Yu , A. L. Rogach , ACS Nano 2013, 7, 11234. 24246067 10.1021/nn405017q

[23] T. Zhang , X. Wang , H. Huang , Y. Liu , Z. Kang , ACS Appl. Mater. Interfaces 2023, 15, 18045. 36989133 10.1021/acsami.3c00036

[24] C. Ji , W. Xu , Q. Han , T. Zhao , J. Deng , Z. Peng , Nano Energy 2023, 114, 108623.

[25] Q.‐L. Chen , C.‐F. Wang , S. Chen , J. Mater. Sci. 2013, 48, 2352.

[26] X. Li , Y. Liu , X. Song , H. Wang , H. Gu , H. Zeng , Angew. Chem., Int. Ed. 2015, 54, 1759. 10.1002/anie.201406836 25212987

[27] Y. Wang , S. Kalytchuk , L. Wang , O. Zhovtiuk , K. Cepe , R. Zboril , A. L. Rogach , Chem. Commun. 2015, 51, 2950. 10.1039/c4cc09589h 25594080

[28] T. Feng , Q. Zeng , S. Lu , X. Yan , J. Liu , S. Tao , M. Yang , B. Yang , ACS Photonics 2018, 5, 502.

[29] S. H. Song , M. Jang , J. Chung , S. H. Jin , B. H. Kim , S. Hur , S. Yoo , Y. Cho , S. Jeon , Adv. Opt. Mater. 2014, 2, 1016.

[30] W. Kwon , Y.‐H. Kim , C.‐L. Lee , M. Lee , H. C. Choi , T.‐W. Lee , S.‐W. Rhee , Nano Lett. 2014, 14, 1306. 24490804 10.1021/nl404281h

[31] T. Zhang , X. Wang , Z. Wu , T. Yang , J. Wang , H. Zhao , H. Huang , Y. Liu , Z. Kang , Appl. Surf. Sci. 2022, 585, 152649.

[32] X.‐Y. Zhang , M.‐P. Zhuo , L.‐S. Liao , Org. Electron. 2021, 96, 106255.

[33] H. Jia , Z. Wang , T. Yuan , F. Yuan , X. Li , Y. Li , Z. Tan , L. Fan , S. Yang , Adv. Sci. 2019, 6, 1900397. 10.1002/advs.201900397 PMC6662328 31380189

[34] J. K. Kim , S. Bae , Y. Yi , M. J. Park , S. J. Kim , N. Myoung , C.‐L. Lee , B. H. Hong , J. Hyeok Park , Sci. Rep. 2015, 5, 11032. 26067060 10.1038/srep11032 PMC4463941

[35] Z. Wang , N. Jiang , M. Liu , R. Zhang , F. Huang , D. Chen , Small 2021, 17, 2104551. 10.1002/smll.202104551 34729915

[36] X. Wang , B. Wang , H. Wang , T. Zhang , H. Qi , Z. Wu , Y. Ma , H. Huang , M. Shao , Y. Liu , Y. Li , Z. Kang , Angew. Chem., Int. Ed. 2021, 133, 12693. 10.1002/anie.202103086 33754433

[37] W. Xu , Q. Han , C. Ji , F. Zeng , X. Zhang , J. Deng , C. Shi , Z. Peng , Small 2023, 19, 2304123. 10.1002/smll.202304123 37649215

[38] J. Liu , Y. Geng , D. Li , H. Yao , Z. Huo , Y. Li , K. Zhang , S. Zhu , H. Wei , W. Xu , J. Jiang , B. Yang , Adv. Mater. 2020, 32, 1906641. 10.1002/adma.201906641 32191372

[39] Y. Zhu , R. Xu , Y. Zhou , Z. Xu , Y. Liu , F. Tian , X. Zheng , F. Ma , R. Alsharafi , H. Hu , T. Guo , T. W. Kim , F. Li , Adv. Opt. Mater. 2020, 8, 2001479.

[40] Y. Cho , S. Pak , B. Li , B. Hou , S. Cha , Adv. Funct. Mater. 2021, 31, 2104239.

[41] A. Hong , J. Kim , J. Kwak , Adv. Opt. Mater. 2020, 8, 2001051.

[42] E. Yao , Z. Yang , L. Meng , P. Sun , S. Dong , Y. Yang , Y. Yang , Adv. Mater. 2017, 29, 1606859. 10.1002/adma.201606859 28394472

[43] J. Chen , J. Wang , X. Xu , J. Li , J. Song , S. Lan , S. Liu , B. Cai , B. Han , J. T. Precht , D. Ginger , H. Zeng , Nat. Photonics 2021, 15, 238.

[44] Z. Chen , Z. Li , Z. Chen , R. Xia , G. Zou , L. Chu , S.‐J. Su , J. Peng , H.‐L. Yip , Y. Cao , Joule 2021, 5, 456.

[45] J. Liu , T. Kong , H. Xiong , Adv. Mater. 2022, 34, 2200152. 10.1002/adma.202200152 35229375

[46] H. Yang , Y. Liu , Z. Guo , B. Lei , J. Zhuang , X. Zhang , Z. Liu , C. Hu , Nat. Commun. 2019, 10, 1789. 30996272 10.1038/s41467-019-09830-6 PMC6470214

[47] Q. Lou , Q. Ni , C. Niu , J. Wei , Z. Zhang , W. Shen , C. Shen , C. Qin , G. Zheng , K. Liu , J. Zang , L. Dong , C. Shan , Adv. Sci. 2022, 9, 2203622. 10.1002/advs.202203622 PMC9596859 36002336

[48] X. Zhang , H. Yang , Z. Wan , T. Su , X. Zhang , J. Zhuang , B. Lei , Y. Liu , C. Hu , Adv. Opt. Mater. 2020, 8, 2000251.

[49] Y. Honmou , S. Hirata , H. Komiyama , J. Hiyoshi , S. Kawauchi , T. Iyoda , M. Vacha , Nat. Commun. 2014, 5, 4666. 25118856 10.1038/ncomms5666

[50] S. Yang , X. Wang , E. Li , X. Liu , J. Hu , J. Liu , J. Photochem. Photobiol. A 2022, 425, 113664.

[51] Q. Dang , B. Zhao , M. Zheng , C. Zhang , R. Yu , S. Qu , H. Jia , Z. Tan , Appl. Phys. Rev. 2024, 11, 011417.

[52] W. Kwon , Y.‐H. Kim , J.‐H. Kim , T. Lee , S. Do , Y. Park , M. S. Jeong , T.‐W. Lee , S.‐W. Rhee , Sci. Rep. 2016, 6, 24205. 27048887 10.1038/srep24205 PMC4822170

[53] X. Gong , J. c. Ostrowski , D. Moses , G. c. Bazan , A. j. Heeger , Adv. Funct. Mater. 2003, 13, 439.

[54] Q. Lou , Q. Ni , C. Niu , J. Wei , Z. Zhang , W. Shen , C. Shen , C. Qin , G. Zheng , K. Liu , J. Zang , L. Dong , C. Shan , Adv. Sci. 2022, 9, 2203622. 10.1002/advs.202203622 PMC9596859 36002336

[55] J. Lee , S. Cheng , S. Yoo , H. Shin , J. Chang , C. Wu , K. Wong , J. Kim , Adv. Funct. Mater. 2015, 25, 361.

[56] Y. Guo , Y. Zhao , Y. Miao , L. Wang , T. Li , H. Wang , B. Xu , J. Yu , J. Mater. Chem. C 2020, 8, 12247.

[57] Q. Wang , Q.‐S. Tian , Y.‐L. Zhang , X. Tang , L.‐S. Liao , J. Mater. Chem. C 2019, 7, 11329.

[58] P. Xiao , J. Huang , Y. Yu , J. Yuan , D. Luo , B. Liu , D. Liang , Appl. Sci. 2018, 8, 1449.

[59] Y. Li , B. Wang , T. Liu , Q. Zeng , D. Cao , H. Pan , G. Xing , ACS Appl. Mater. Interfaces 2022, 14, 3284. 34989549 10.1021/acsami.1c21000

[60] G. Xu , R. Xue , S. J. Stuard , H. Ade , C. Zhang , J. Yao , Y. Li , Y. Li , Adv. Mater. 2021, 33, 2006753. 10.1002/adma.202006753 33634532

[61] P. Gao , Y. Zhang , P. Qi , S. Chen , Adv. Opt. Mater. 2022, 10, 2202066.

[62] J. Chang , M. C. Gwinner , M. Caironi , T. Sakanoue , H. Sirringhaus , Adv. Funct. Mater. 2010, 20, 2825.

[63] Z. Chen , Z. Li , T. R. Hopper , A. A. Bakulin , H.‐L. Yip , Rep. Progr. Phys. 2021, 84, 046401. 10.1088/1361-6633/abefba 33730709

[64] H. Shen , Q. Gao , Y. Zhang , Y. Lin , Q. Lin , Z. Li , L. Chen , Z. Zeng , X. Li , Y. Jia , S. Wang , Z. Du , L. S. Li , Z. Zhang , Nat. Photonics 2019, 13, 192.

[65] R. W. Jaggers , R. Chen , S. A. F. Bon , Mater. Horiz. 2016, 3, 41.

[66] J. Zhao , C. Zheng , Y. Zhou , C. Li , J. Ye , X. Du , W. Li , Z. He , M. Zhang , H. Lin , S. Tao , X. Zhang , Mater. Horiz. 2019, 6, 1425.

