# Supplementary material for: Binary Host‐induced Exciplex Enabled High Color‐Rendering Index of 94 for Carbon Quantum Dot‐Based White Light‐Emitting Diodes
Source: Adv Sci (Weinh). 2024 Jun 13;11(30):2404485. doi: 10.1002/advs.202404485 (PMC11321674; doi:10.1002/advs.202404485)
Supplement: Supplementary file 1 — Supporting Information [file ADVS-11-2404485-s001.docx]

Supporting Information

**Binary Host-induced Exciplex enabled High Color-Rendering Index of 94 for Carbon Quantum Dot-based White Light-Emitting Diodes**

Renjing Chen,^[a]^ Zhibin Wang*,^[a]^ Qian Teng,^[c]^ Chenhao Li,^[c]^ Jinsui Li,^[c]^ Lingwei Zeng,^[d]^ Ruidan Zhang,^[a]^ Feng Huang,^[a]^ Lei Lei,^[e]^ Fanglong Yuan*,^[c]^ Daqin Chen*^[a][b]^

**Experiments**

**Materials:** 1,3,5-tris(2-N-phenylbenzimidazolyl)-benzene (TPBi), poly[bis(4-phenyl)(2,4,6-trimethylphenyl)amine] (PTAA), [2,4,6-Tris[3-(diphenylphosphinyl) phenyl]-1,3,5-Triazine] (PO-T2T) and lithium fluoride (LiF), (poly(9,9-dioctylfluorenyl-2,7-diyl)-alt-(4,4′-(*N*-(4-butylphenyl) (TFB), [poly(bis(4-phenyl)(4-butylphenyl)amine)] (Poly-TPD), poly(9-vinylcarbazole) (PVK) and Poly(2,3-dihydrothieno-1,4-dioxin)-poly(styrenesulfonate) (PEDOT:PSS) were purchased from Xi’an Polymer Light Technology Corp. Anhydrous chlorobenzene (CB, 99^+^%), and Dichloromethane (CH_2_Cl_2_, ≥ 99.9% , GC residue analysis) were purchased from Beijing Innochem Science & Technology Corp. Methanol (CH_3_OH) and cyclohexane (C_6_H_12_) were purchased from Shanghai Macklin Biochemical Technology Corp. Anhydrous ethyl alcohol (≥ 99.7%) was purchased from XiLong Scientific Corp. Clovers were picked on the campus of Fujian Normal University.

**Synthesis of CQDs:** Take fresh Oxalis corniculata L. and wash its surface thoroughly with water. Then, place it in a drying oven to facilitate dehydration. Measure out 10 g of the dried clover and immerse it in 50 ml of anhydrous ethanol. Stir this mixture for 24 h, and then filter the residue through filter paper to obtain a clarified green solution. Transfer 10 ml of the obtained precursor along with 2.5 ml of cyclohexane into a 25-ml reaction vessel. Place this vessel in a drying oven and subject it to a reaction at 150°C for 6 h. After the reaction, allow the mixture to cool naturally to room temperature and retrieve the crude product. Filter the product using a 220 nm PVDF membrane. For further purification, use a silica gel chromatography column with a mixture of dichloromethane and methanol as the eluent. Adjust the ratio of dichloromethane to methanol to optimize elution efficiency. Finally, freeze-dry the product and store it at 4°C for future use.

**Device Fabrication:** The preparation methods of Device-Ⅰ and Ⅱ are the same. The indium tin oxide (ITO) coated glass substrates were ultrasonically cleaned with soapy water, water, deionized water, acetone, anhydrous ethanol, and isopropanol for 20 minutes, respectively. The surface liquid of ITO substrate was blown dry with nitrogen gas, annealed at 150°C for 10 minutes, and then transferred to UV-Ozone treatment for 15 minutes before use. The hole transport layer (HTL) was prepared by spin-coating method by spin-coating the filtered PEDOT:PSS (30 nm) onto the ITO substrate at 4000 rpm for 40 s and annealing at 150 °C for 15 minutes. The ITO substrate coated with PEDOT:PSS was then transferred to a glove box filled with N_2_. The emission layer (50 nm) precursor solutions were prepared by mixing the same concentration (6 mg mL^-1^) of CQDs with PTAA, TFB, Poly-TPD solutions and PO-T2T solutions. The emissive layer was prepared by spin-coating the precursor solution onto the ITO substrate at 4000 rpm for 40 s and then annealing at 80°C for 10 minutes in a nitrogen-filled glove box. After the emissive layer was prepared, PO-T2T (40 nm), LiF (1 nm) and Al (100 nm)/TPBi (40 nm), LiF (1 nm) and Al (100 nm) were deposited by thermal evaporation under high vacuum conditions of less than about 2 × 10^-4^ Pa. The effective area of the device was determined to be 4 mm^2^.

**Characterization:** TEM images of CQDs were obtained on a JEOL JEM-2010 at an acceleration voltage of 200 kV. AFM images were recorded on a Bruker Dimension icon. The XPS were measured by an ESCALab220i-XL multifunction electron spectrometer. Steady-state photoluminescence (PL) spectra and Time-resolved PL spectra were measurements a FLS1000 ﬂuorescence lifetime spectrometer (Edinburgh Instruments Ltd.). Ultraviolet–visible (UV–Vis) absorption spectra were obtained on a Techcomp UV1101 Ⅱ Spectrophotometer. CQDs-based WLEDs were tested for optoelectronic properties in an unencapsulated nitrogen-filled glove box. EL characteristic curves and current density-voltage luminance (J-V-L), external quantum efficiency-voltage (EQE-V) of CQDs-based WLEDs were collected using a computer-controlled Keithley 2400 source measurement unit, and with a fiber-optic integrated sphere and a photonic multichannel analyzer PMA-12.

**Figure S1.** (a) TEM image of CQDs. (b) (100) planes of graphite with a distance of 0.21 nm. Statistical analyses were carried out with Digital MicroGraph3.9. (c) TEM image of CQDs.

**Figure S2.** the X-ray powder diffraction pattern of CQDs.

**Figure S3.** (a) Full-survey X-ray photoelectron spectroscopy (XPS) spectrum of CQDs. (b) High-resolution N 1s XPS spectra of CQDs. (c) High-resolution O 1s XPS spectra of CQDs.

**Figure S4.** Fourier transform infrared spectrum of CQDs.

**Figure S5.** Raman spectra of CQDs.


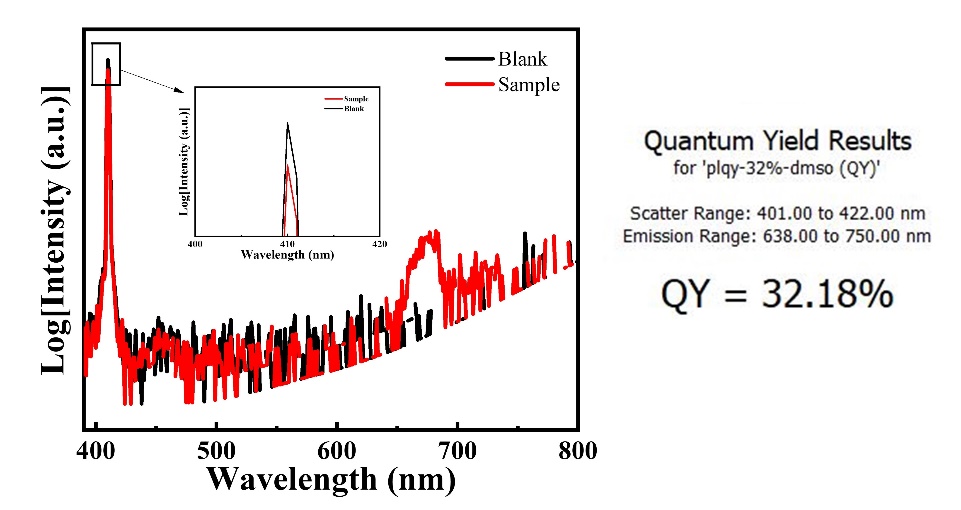


**Figure S6.** The absolute PLQY of CQDs in DMSO solution under the excitation wavelength of 410 nm.


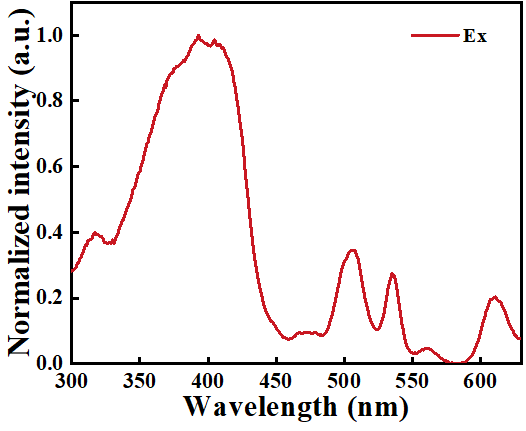


**Figure S7.** The excitation spectra of CQDs.


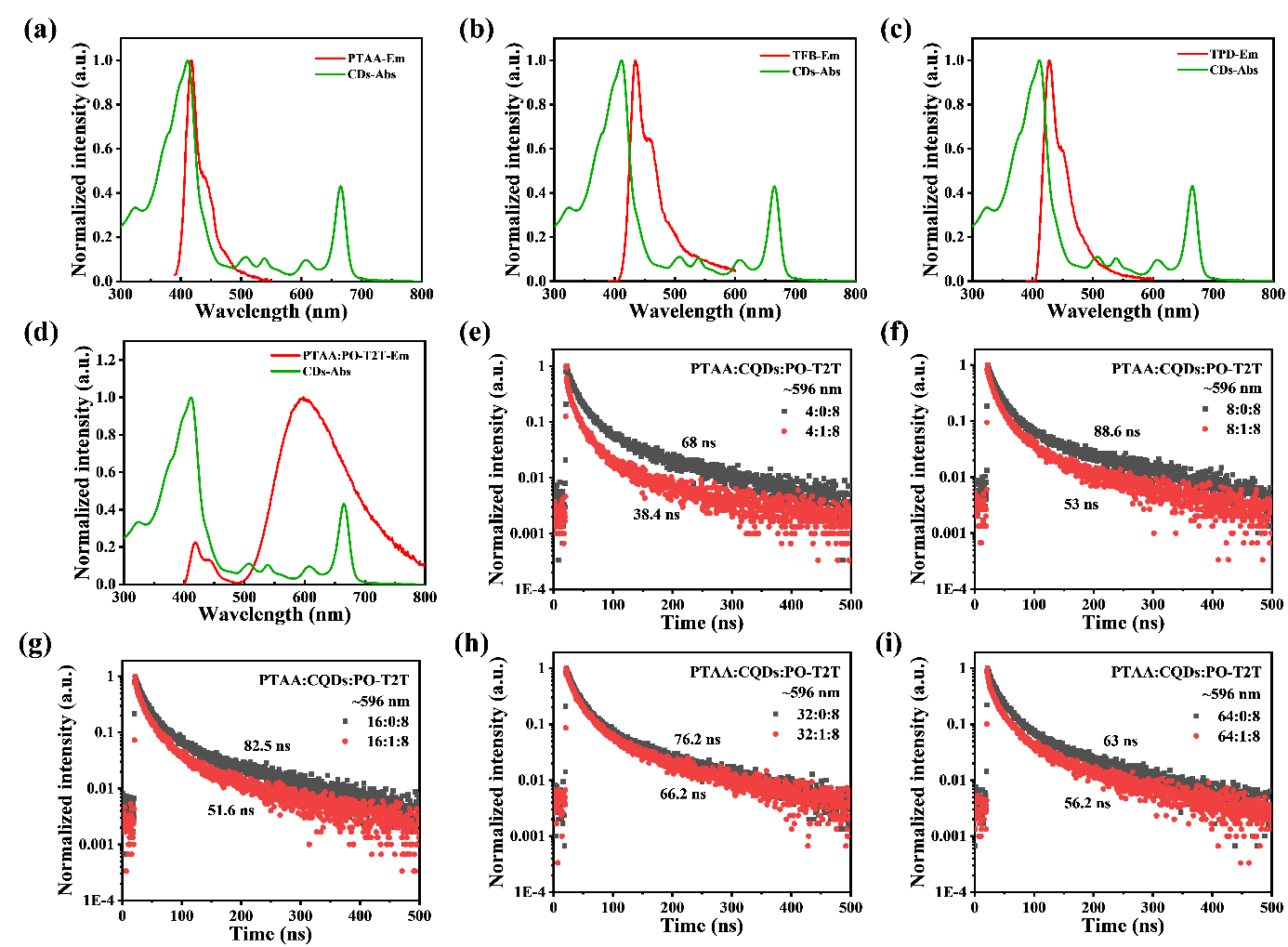


**Figure S8.** (a) The absorption spectrum of CQDs and PTAA of PL spectrum. (b) The absorption spectrum of CQDs and TFB of PL spectrum. (c) The absorption spectrum of CQDs and TPD of PL spectrum. (d) The absorption spectrum of CQDs and PTAA:PO-T2T of PL spectrum. PL lifetimes of PTAA:CQDs:PO-T2T thin films with molar ratios of (e) 4:0:8 and 4:1:8, (f) 8:0:8 and 8:1:8, (g) 16:0:8 and 16:1:8, (h) 32:0:8 and 32:1:8, and (i) 64:0:8 and 64:1:8 with probing wavelength at 596 nm.

**Figure S9.** The molecular structure of (a) TFB, and (b) Poly-TPD.

**Figure S10.** The PL spectra of (a) TFB:CQDs, (b) TFB:PO-T2T, and (c) TFB:CQDs:PO-T2T thin films.

**Figure S11.** The PL spectra of (a) Poly-TPD:CQDs, (b) Poly-TPD:PO-T2T, and (c) Poly-TPD:CQDs:PO-T2T thin films.

**Figure S12.** The absorption spectra of (a) PTAA:CQDs, (b) TFB:CQDs, (c) Poly-TPD:CQDs, (d) PTAA:PO-T2T, (e) TFB:PO-T2T, (f) Poly-TPD:PO-T2T, (g) PTAA:CQDs:PO-T2T, (h) TFB:CQDs:PO-T2T, and (i) Poly-TPD:CQDs:PO-T2T thin films.


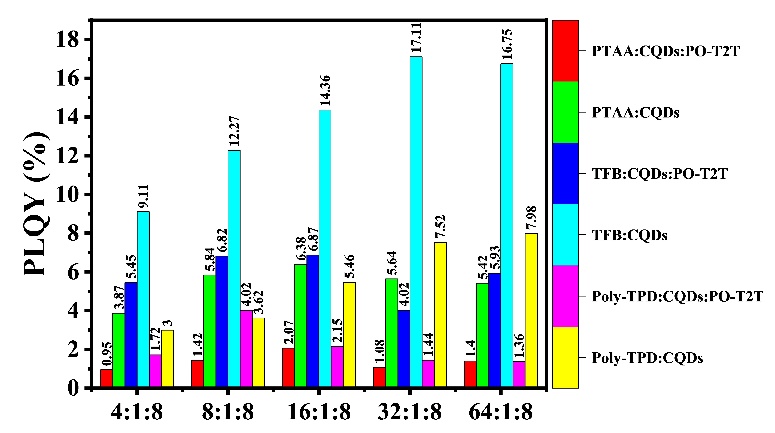


**Figure S13.** The PLQY of thin films.


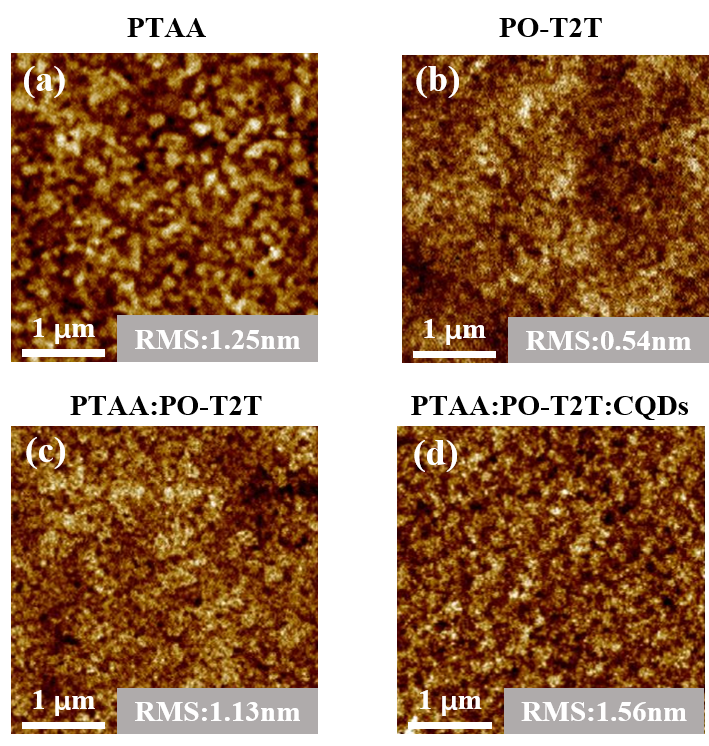


**Figure S14.** The AFM images of (a) PTAA, (b) PO-T2T, (c) PTAA:PO-T2T, and (d) PTAA:PO-T2T:CQDs thin films.

**Table S1.** The Electroluminescent performance parameters of CQDs-based WLEDs.

| Emissive layer | CRI | CCT  (K) | L_max_  (cd m^-2^) | (X, Y) | V_on_  (V) | Ref. |
| --- | --- | --- | --- | --- | --- | --- |
| CQDs | 82 | NA | 35 | (0.40, 0.43) | 6.0 | [1] |
| CQDs | NA | NA | 90 | (0.318,0.320) | 4.6 | [2] |
| TCTA:CQDs:TPBi | NA | NA | 29 | (0.2894, 0.3351) | NA | [3] |
| MCBF-CQDs | NA | NA | 2050 | (0.30, 0.33) | 3.9 | [4] |
| MADN:Rubrene/CQDs | NA | NA | NA | (0.34, 0.37) | 12.0 | [5] |
| PVK:CQDs | 83 | 7694 | 455.2 | NA | NA | [6] |
| R-EGP-CQDs-NMe_2_/-NEt_2_/-NPr_2_:PVK | NA | NA | 5909 | (0.388, 0.309) | 3.0 | [7] |
| GQD/TMPE/LiTf | NA | NA | NA | (0.38, 0.36) | 7.0 | [8] |
| TFB:Y-CQDs | 76 | NA | 2422 | (0.33, 0.33) | 4.1 | [9] |
| PVK:CQDs | NA | NA | 598 | (0.33, 0.33) | 2.8 | [10] |
| mCP:PVK:WCQDs | NA | NA | NA | (0.379, 0.414) | 6.0 | [11] |
| phCN | NA | 3700 | 188 | (0.44, 0.52) | 3.2 | [12] |
| CQDs | NA | NA | 30.01 | (0.3627, 0.4139) | 7.0 | [13] |
| CQDs:PO-T2T:PTAA | 94.33 | 4976 | 2375.5 | (0.34, 0.32) | 2.8 | This work |
| CQDs:PO-T2T:TFB | 85.32 | 5503 | 1726.7 | (0.27, 0.30) | 4.6 | This work |
| CQDs:PO-T2T:Poly-TPD | 90 | 3687 | 1249 | (0.37, 0.30) | 4.6 | This work |

**Figure S15.** The EL spectrum of (a) TFB:CQDs/PO-T2T, (b) Poly-TPD:CQDs/PO-T2T, (c) TFB:CQDs:PO-T2T/TPBi, and (d) Poly-TPD:CQDs:PO-T2T/TPBi.

**Figure S16.** (a) Current density–voltage (J-V) curve of Device-Ⅰ. (b) Luminance–voltage (L-V) curve of Device-Ⅰ. (c) EQE-voltage curves of Device-Ⅰ. (d) J-V curve of Device-Ⅱ. (e) L-V curve of Device-Ⅱ. (f) EQE-voltage curves of Device-Ⅱ.

**Figure S17.** The operational lifetime of CQDs-based WLEDs at an initial luminance of ~100 cd m^-2^.

**Figure S18.** Histograms of (a) peak luminance and (b) peak EQE of CQDs-based WLEDs.

**REFERENCES**

[1] F. Wang, *Chem. Commun.* **2011**, 3.

[2] X. Zhang, Y. Zhang, Y. Wang, S. Kalytchuk, S. V. Kershaw, Y. Wang, P. Wang, T. Zhang, Y. Zhao, H. Zhang, T. Cui, Y. Wang, J. Zhao, W. W. Yu, A. L. Rogach, *ACS Nano* **2013**, *7*, 11234.

[3] S. Do, W. Kwon, Y.-H. Kim, S. R. Kang, T. Lee, T.-W. Lee, S.-W. Rhee, *Adv. Opt. Mater.* **2016**, *4*, 276.

[4] F. Yuan, Z. Wang, X. Li, Y. Li, Z. Tan, L. Fan, S. Yang, *Adv. Mater.* **2017**, *29*, 1604436.

[5] K. Qian, H. Li, B. Zheng, F. Chang, Q. Lei, G. Han, Y. Song, S. Liu, Y. Wei, *Org. Electron.* **2017**, *51*, 314.

[6] J. Xu, Y. Miao, J. Zheng, H. Wang, Y. Yang, X. Liu, *Nanoscale* **2018**, *10*, 11211.

[7] H. Jia, Z. Wang, T. Yuan, F. Yuan, X. Li, Y. Li, Z. Tan, L. Fan, S. Yang, *Adv. Sci.* **2019**, *6*, 1900397.

[8] K. Chu, J. R. Adsetts, S. He, Z. Zhan, L. Yang, J. M. Wong, D. A. Love, Z. Ding, *Chem. Eur. J.* **2020**, *26*, 15892.

[9] Z. Wang, N. Jiang, M. Liu, R. Zhang, F. Huang, D. Chen, *Small* **2021**, *17*, 2104551.

[10] X. Wang, B. Wang, H. Wang, T. Zhang, H. Qi, Z. Wu, Y. Ma, H. Huang, M. Shao, Y. Liu, Y. Li, Z. Kang, *Angew. Chem.* **2021**, *133*, 12693.

[11] X. Zhou, K. Yi, Y. Yang, G. Xie, X. Ji, Z. He, *Nano Res.* **2022**, *15*, 9470.

[12] Y. Wang, K. Wang, F. Dai, K. Zhang, H. Tang, L. Wang, J. Xing, *Nat. Commun.* **2022**, *13*, 6495.

[13] X. Zhang, L. Zeng, T. Sun, X. Xue, B. Wei, *Phys. Chem. Chem. Phys.* **2022**, *24*, 26511.
